# Surface alterations of the MicroMega One RECI reciprocating instrument under two kinematics: A scanning electron microscopy evaluation

**DOI:** 10.4317/jced.63988

**Published:** 2026-09-15

**Authors:** Kevin Denis Lavado-Cuicapusa, Maria Mihaela Iuga, Carlos Mendiola-Aquino, Antonio Ernesto Denegri-Hacking, Carlos Garcia-Puente, Alejandro Jaime, Mario Casaretto-Gamonal

**Affiliations:** 1 Department of Stomatological Clinic, Cayetano Heredia Peruvian University, Lima, Perú; 2 Faculty of Health Sciences, Private University of Tacna, Tacna, Perú; 3 Maimonides University, Buenos Aires, Argentina

**Keywords:** Endodontics, reciprocating motion, adaptive reciprocation, nickel-titanium instruments, scanning electron microscopy.

## Abstract

**Background:**

This ex vivo study compared surface alterations of MicroMega One RECI 25/.06 instruments after use under two reciprocating kinematics: Adaptive Intelligent Reciprocation (ARI) and the manufacturer’s standard reciprocating motion (NOR).

**Materials and Methods:**

Twenty new One RECI instruments were first examined by scanning electron microscopy (SEM) to establish their factory-new baseline condition (FAB). The same instruments were then randomly allocated to ARI (n=10) or NOR (n=10). Each file was used to prepare three mesial canals of extracted mandibular molars with moderate curvature and was subsequently re-examined by SEM. Surface defects were scored by instrument segment and classified according to predefined operational criteria as dull edges, deformed flutes, microcracks, craters, or tip deformation. Data were analyzed using descriptive statistics, non-parametric tests, and Poisson regression modeling.

**Results:**

Dull edges and deformed flutes were the most frequently observed defects. Factory-new instruments showed minimal baseline alterations, whereas used instruments exhibited significantly higher defect counts. In the unadjusted pairwise comparison, ARI and NOR did not differ significantly. However, Poisson regression suggested a higher expected number of defects in the NOR group compared with ARI.

**Conclusions:**

Within the limitations of this ex vivo paired pre-post study, repeated use increased surface alterations in One RECI instruments. ARI showed a tendency toward fewer defects than NOR, although the between-kinematic comparison should be interpreted cautiously because of the exploratory sample size. These findings support limiting instrument reuse and suggest that adaptive reciprocation may reduce cumulative surface damage.

## Introduction

Engine driven nickel-titanium (NiTi) instruments are essential in endodontics due to their high flexibility and shape memory, which enable safer preparation of curved root canals compared with stainless steel hand files [1,2]. However, surface damage, which may progress to instrument separation via cyclic or torsional fatigue, remains a major concern for clinicians. Various technological strategies have been developed to address these limitations, including design modifications, altered cross sections, different kinematics, and thermal treatments [3-6].

Reciprocating motion, first introduced by Yared [7], alternates a counterclockwise cutting rotation with a shorter clockwise release. This pattern reduces screwing in and torsional stress, supporting single-file techniques for root canal preparation. Previous studies have shown that reciprocation provides higher cyclic fatigue resistance and greater safety compared with continuous rotary motion, especially in constricted or severely curved canals [8,9].

The MicroMega One RECI system (Coltene, Lezennes, France) uses C Wire alloy and electropolishing to improve flexibility, cyclic fatigue resistance, and surface quality [10]. Nevertheless, like other NiTi instruments, One RECI files remain susceptible to surface defects, such as microcracks, craters, dull edges, deformed flutes, and tip deformation, both from manufacturing and clinical use [11]. Similar defects have been documented in systems such as WaveOne, Reciproc, and Reciproc Blue [12].

Recently, new reciprocating kinematics have emerged aiming to further reduce stress on NiTi files. Motion parameters strongly influence the frequency of stress-relief events per cycle; more frequent unloading events may slow fatigue accumulation and delay instrument failure. The Endo Radar Pro motor (Woodpecker, Guilin, China) features an Adaptive Intelligent Reciprocation (ARI) mode that dynamically modifies reciprocation angles according to canal resistance, typically 90°CCW / 30°CW, reducing to 40°/20° when torque reaches 0.4 N·cm (500 rpm), to minimize stress accumulation.

To date, no study has evaluated whether torque-responsive adaptive reciprocation affects the microscopic surface integrity of One RECI files. Prior work has focused on conventional reciprocation or comparisons with other systems, leaving a gap regarding adaptive kinematics. Biomechanical theories propose that increased unloading frequency may reduce cumulative stress, but this has not been validated via topographic assessment. Given that ARI was only recently commercialized and its algorithm is proprietary, laboratory-based SEM evidence is lacking. This study addresses this gap by asking: Does adaptive motion reduce real structural damage in One RECI files compared with standard manufacturer motion? The null hypothesis is that there is no significant difference in the type or frequency of surface defects between the two kinematic protocols.

## Materials and Methods

*Study design and instrument allocation*

This *ex vivo* experimental study used a paired pre-post within-instrument design. Twenty new MicroMega One RECI NiTi instruments, size 25/.06, were included. All instruments were first examined by SEM in their factory-new condition to establish baseline surface status, defined as the FAB condition. After baseline imaging, the same instruments were randomly allocated to one of two experimental kinematic groups: Adaptive Intelligent Reciprocation, ARI, n=10, or manufacturer’s standard reciprocation, NOR, n=10. After canal preparation, each instrument was re-examined by SEM. Thus, each file served as its own baseline control, while the post-use comparison between ARI and NOR was considered the primary exploratory intergroup comparison.

In ARI mode, the Endo Radar Pro motor used the adaptive reciprocation program, 90° counterclockwise and 30° clockwise, reducing to 40° counterclockwise and 20° clockwise when torque reached 0.4 N·cm, at 500 rpm. In the NOR group, instruments were used according to the manufacturer’s standard reciprocating program, 170° counterclockwise and 60° clockwise, with the same torque and speed settings.

*Sample size justification*

The sample size (n=20) was chosen to assure >80% power (α*=*0.05) for the paired pre-post comparison, assuming a minimum relevant increase of ~1 defect (SD≈1.5; dz≈0.67). The ARI versus NOR comparison (10 instruments per group) was pre-specified as exploratory; given the small observed difference (RR≈1.75), this contrast is underpowered, consistent with the observed non-significant result.

*Canal preparation*

Sixty mesial canals from extracted human mandibular molars were selected. Teeth had been extracted for non-endodontic reasons and were anonymized before use. Inclusion criteria were fully formed apices, absence of previous endodontic treatment, absence of root resorption or cracks, and moderate canal curvature between 10° and 30° according to Schneider’s method.

To standardize specimen length, crowns were removed at the cementoenamel junction, and roots were adjusted to a length of 17 mm before access preparation. Standard access cavities were prepared using high-speed burs under water cooling. Apical patency was confirmed with a #10 K-file, and working length was established with a #15 K-file inserted until visible at the apical foramen and then reduced by 1 mm. A glide path was confirmed with a #15 K-file before mechanical preparation.

Each One RECI instrument was used to prepare three mesial canals. Therefore, 30 canals were prepared in the ARI group and 30 canals in the NOR group. Instrumentation was performed using gentle in-and-out pecking movements until working length was reached, with a maximum of three pecks per canal. After each pecking sequence, the file was removed, cleaned, and the canal was irrigated with 5 mL of 5.25% sodium hypochlorite. After canal preparation, a final rinse with 2 mL of 17% EDTA was performed.

*SEM analysis*

Each instrument was examined before use and after three canal preparations using a FEI Inspect S50 scanning electron microscope at magnifications ranging from 100× to 2000×. Three standardized regions were assessed in each instrument: Apical third, 1-3 mm from the tip; middle third, 6-8 mm from the tip; and cervical third, 11-13 mm from the tip. After instrumentation, each file was immediately rinsed with distilled water to remove organic debris and irrigant residues. The instruments were gently brushed under running water for 3 min, ultrasonically cleaned in distilled water for 5 min, dried with oil-free compressed air, and stored individually in labeled sterile tubes. No chemical agent capable of altering the metallic surface was applied before SEM analysis.

Surface defects were recorded according to predefined operational criteria. Dull edges were defined as loss of sharpness or rounding of the cutting edge without visible metal displacement. Deformed flutes were defined as distortion, unwinding, flattening, or irregular deformation of the flute architecture. Microcracks were defined as linear discontinuities or fissure-like defects on the metal surface. Craters were defined as localized pits, depressions, or material loss on the instrument surface. Tip deformation was defined as bending, blunting, distortion, or irregular alteration of the instrument tip morphology.

Micrographs were coded before scoring. Defects were recorded by instrument segment and defect type. When multiple defects of the same type were observed in the same segment, they were counted separately only if they were clearly distinguishable and spatially separated. Disagreements or uncertain findings were resolved by consensus after re-evaluation of the micrographs.

*Statistical analysis*

Data were analyzed using R version 4.3.2. Descriptive statistics were calculated as frequencies, means, medians, standard deviations, and ranges. The Shapiro-Wilk test was used to evaluate the distribution of defect counts. Because the data were non-normally distributed, non-parametric tests were applied. The Wilcoxon signed-rank test was used to compare baseline factory-new measurements with post-use measurements within the same instruments. The Mann-Whitney U test was used for the exploratory post-use comparison between ARI and NOR groups. For descriptive purposes, Kruskal-Wallis testing with Dunn’s post hoc correction was also used to compare FAB, ARI, and NOR conditions. A Poisson regression model was fitted to estimate the association between kinematic mode, use condition, and instrument segment with total defect counts. Statistical significance was set at α=0.05.

## Results

*Descriptive findings*

New factory control instruments (FAB group) showed virtually no surface damage (mean total defects per file=0.15, SD=0.48), whereas instruments used under the conventional reciprocation program (NOR group) exhibited the highest defect counts (mean=1.63, SD=1.47). Files used with the adaptive mode (ARI group) had intermediate defect levels (mean=0.93, SD=1.14) (Table 1).

The most frequently observed defect types overall were dull cutting edges (25.8% of all recorded defects) and deformed flutes (18.3%), while tip deformation was the least common. Figure 1 shows the distribution of total defects per instrument in each group and by condition (new vs. used). The FAB group consistently had no or minimal defects per file, whereas the NOR group had the greatest number and the ARI group was intermediate. Instruments subjected to three uses accumulated clearly more surface damage than new instruments.

*Inferential statistics*

The data failed normality (Shapiro-Wilk W=0.669, *p*<0.001), necessitating non-parametric analysis. A Kruskal-Wallis test indicated a significant difference in total defect counts across the three groups (χ²=38.22, df=2, *p*<0.001). Dunn’s pairwise comparisons showed both ARI and NOR groups had significantly more defects than the FAB group (adjusted *p*<0.001), whereas ARI vs. NOR did not differ significantly (*p*=0.18). The

combined used files (ARI + NOR) had significantly more defects than unused instruments (Wilcoxon W=813.5, *p*<0.001) (Table 2).

In the exploratory Poisson regression model, after accounting for instrument segment and use condition, FAB instruments showed significantly fewer defects than used instruments (β=-1.83, *p*<0.001). The NOR group showed a higher expected defect count than ARI (β=0.56, *p*=0.018), corresponding to an estimated increase of approximately 75% in expected defect counts. However, because the unadjusted ARI versus NOR pairwise comparison was not statistically significant, this model-based finding should be interpreted as exploratory and hypothesis-generating rather than confirmatory (Figure 2).

Defect distribution patterns: Qualitative SEM analysis revealed damage was not uniform along file length. Dulling of cutting edges predominated in the middle third, whereas microcracks were more frequent in the apical third. The cervical third generally showed the fewest alterations. In terms of group differences, NOR had the highest incidence of dull edges and deformed flutes, followed by ARI; FAB showed almost no defects. Microcraters and minor tip damage occurred mainly in the used groups (especially NOR) (Figures 3,4). Collectively, results show repeated clinical use leads to greater and more varied surface alterations, and that adaptive reciprocation tended to produce slightly less damage than standard reciprocation.

Supplementary 1 (https://www.medicinaoral.com/medoralfree01/63988/Suppl1.pdf) displays the distribution of total surface defects by group: FAB showed minimal dispersion; ARI exhibited a wider spread, with some files reaching up to five alterations; NOR had the greatest variability, often with two or more defects. Representative SEM micrographs before and after use Supplementary 1 (https://www.medicinaoral.com/medoralfree01/63988/Suppl1.pdf) confirm these patterns. Factory new files Supplementary 1 (https://www.medicinaoral.com/medoralfree01/63988/Suppl1.pdf) had smooth surfaces; after three uses, NOR files Supplementary 1 (https://www.medicinaoral.com/medoralfree01/63988/Suppl1.pdf) showed multiple defects, including craters, dull edges, deformed flutes, and tip deformation, while ARI files Supplementary 1 (https://www.medicinaoral.com/medoralfree01/63988/Suppl1.pdf) had minimal alterations, mainly dull edges, slight flute deformation, and isolated microcracks.

## Discussion

The impact of reciprocating motion kinematics on stress distribution, surface degradation, and fracture susceptibility of nickel-titanium (NiTi) instruments remains a critical area of investigation in endodontics. 

Previous studies have established that reciprocation motion parameters play a decisive role in the mechanical behavior of NiTi files. In particular, reducing reciprocation angles substantially increases the frequency of stress-relief events during each cycle [13-15]. 

For instance, larger reciprocation angles such as 180°/30° produce approximately three unloading phases per cycle, whereas smaller angles such as 70°/30° may generate up to nine. Increasing the number of unloading phases delays fatigue accumulation and may prolong the instrument’s resistance to fracture, as demonstrated by recent mechanical evaluations of thermal-treated NiTi systems [16]. This mechanism helps explain the trend toward fewer surface alterations observed in the ARI group in the present study, supporting the premise that adaptive kinematics and reduced angular displacement distribute mechanical loads more evenly and mitigate cumulative structural damage.

In the present study, we evaluated the surface topography of the MicroMega One RECI file after three uses under two kinematic conditions: The manufacturer-recommended conventional reciprocation (NOR) and the adaptive reciprocation mode (ARI) integrated into the Endo Radar Pro motor. Although the difference in defect frequency between the two groups was not statistically significant, a discernible trend toward fewer alterations in the ARI group was observed. From a biomechanical perspective, ARI operates with an adaptive torque threshold of 0.4 N·cm, reinforcing the notion that lower angular deflection and increased flexibility may help reduce superficial damage. Under these conditions, the reduction of reciprocation angles directly influences the modulation of mechanical stress by increasing the frequency of unloading (stress-relief) events along the file [17].

In our specimens, blunt edges, deformed flutes, microcracks, and craters were the predominant findings, features typically associated with mixed-mode failure combining torsional load and cyclic fatigue, consistent with previously described patterns of surface damage reported by Valois *et al*. [18], Pérez *et al*. [19], and Plotino *et al*. [20]. Notably, in the ARI group, these alterations appeared more evenly distributed along the instrument surface, contrasting with studies describing non-homogeneous stress concentration, where the apical third experiences the highest combination of flexion and torsion, the middle third is exposed to transitional loading, and the coronal third undergoes predominantly lateral friction. The more homogeneous stress distribution generated during ARI motion, likely driven by the increased frequency of stress-relief events, may explain the trend toward lower surface damage observed in the present study. These findings provide clinicians with relevant insight into how kinematic configurations, combined with thermally treated NiTi alloys, may offer advantages in complex or highly curved canals by mitigating premature structural deterioration of the instrument.

Our findings are consistent with those of Howait [3], who reported surface defects in 30% of Reciproc files after a single clinical use, despite strict adherence to manufacturer protocols. Their observations, mainly disrupted cutting blades and surface holes, underscore the susceptibility of NiTi instruments to subcritical, mixed-mode failure mechanisms involving both torsional stress and cyclic fatigue, even after limited use. In our study, One RECI files used in NOR mode exhibited an average of 1.63 defects per instrument, with blunt edges and deformed flutes being the predominant alterations. These findings support the notion that increasing the number of uses correlates with heightened surface degradation. Such approaches may provide clinicians with clearer guidance regarding the approximate number of safe uses and the mechanical limits of NiTi instruments exposed to repeated loading.

Similarly, Zanza *et al*. [9] demonstrated that after four simulated clinical uses, One RECI and other reciprocating systems exhibited cumulative damage, particularly in the apical third. While our protocol involved a lower number of cycles, wear patterns were also identified in this region; however, the overall distribution of defects in our specimens was more homogeneous across the apical, middle, and cervical thirds, except for significantly fewer defects in the cervical third (β=-2.81; *p*<0.001). This discrepancy may be related to the incorporation of adaptive kinematics in one of our groups, which increases the frequency of stress-relief events, as well as potential differences in instrument design, heat treatment, and the lower cumulative mechanical load applied in our experimental protocol.

Chopra and Ballal [21] compared WOG and Jizai files after three uses in simulated curved canals and reported more pronounced defects in the coronal and middle thirds. In contrast, our findings in natural human teeth revealed a different distribution pattern. Microcracks were most frequently observed in the apical third, whereas dull cutting edges predominated in the middle third; the cervical third consistently exhibited significantly fewer alterations (β=-2.81; *p*<0.001). This pattern aligns with the biomechanical expectation that stresses concentrate toward the apical and middle regions during canal preparation. Moreover, when comparing kinematic modes, the ARI group displayed a more homogeneous distribution of defects along the instrument, while the NOR group presented a higher incidence of dull edges and deformed flutes, consistent with greater mechanical load. These observations not only contrast with the defects reported by Chopra and Ballal [21], but also demonstrate that, in our dataset, surface damage can be meaningfully categorized by canal third, providing clearer insight into how specific stress environments influence defect type and prevalence.

This difference underscores the possible advantage of using adaptive reciprocation with real anatomical variability, as well as the influence of torque-responsive angular reduction in preserving structural integrity.

Finally, Kiraz *et al*. [22] identified a direct relationship between repeated use and increased surface damage, recommending single-use protocols to mitigate fracture risk. While our study limited reuse to three cycles, the significantly lower defect incidence in the ARI group reinforces the relevance of adaptive kinematics in reducing structural compromise. Notably, even with limited reuse, visible alterations occurred across all instruments, emphasizing the importance of cautious clinical reuse and the potential benefits of intelligent kinematic modulation.

*Limitations*

This study has several limitations. First, although the paired pre-post design allowed each instrument to serve as its own baseline control, the intergroup comparison between ARI and NOR included only 10 instruments per group and should therefore be interpreted as exploratory. 

Second, the *ex vivo* design cannot fully reproduce the complexity of clinical instrumentation, including variations in operator pressure, canal anatomy, intracanal debris, irrigant dynamics, and repeated sterilization cycles. 

Third, SEM analysis provides detailed surface information but does not allow evaluation of subsurface crack propagation, metallurgical phase transformation, or internal structural fatigue. 

Fourth, torque peaks were not continuously recorded during instrumentation; therefore, direct correlations between real-time mechanical stress and surface defect formation could not be established. 

Finally, if examiner blinding was not feasible during image scoring, potential measurement bias cannot be completely excluded. 

These limitations should be considered when interpreting the apparent reduction in surface alterations observed with ARI.

*Clinical implications*

The present findings suggest that torque-responsive adaptive reciprocation may attenuate microscopic surface damage in nickel-titanium instruments. Although differences did not reach statistical significance, the adaptive mode consistently exhibited fewer alterations and a more homogeneous distribution of defects. This supports the pragmatic notion that motion parameters, in combination with thermally treated alloys, can influence mechanical load patterns and potentially extend the safe lifespan of files. 

Clinicians operating in curved or complex anatomies may therefore consider kinematic settings that promote stress-relief events to minimize cumulative structural degradation. These findings suggest that adaptive reciprocation may be particularly advantageous during retreatment cases or canal anatomy with curvature complexity exceeding 25°, where cumulative stress and microdamage become more clinically relevant.

Although the difference between kinematic modes did not reach statistical significance, the direction and magnitude of the effect are clinically meaningful because surface degradation is a cumulative process and even subtle microscopic changes may accelerate fatigue progression over multiple uses.

*Future research*

Further investigations should incorporate dynamic torque monitoring, fractographic evaluation, and fatigue-to-failure testing to clarify how stress accumulation translates into crack initiation and propagation. Studies in real clinical scenarios or CBCT-validated anatomies, as well as comparative trials among different adaptive platforms, are warranted to determine whether kinematic modulation consistently improves safety and reliability under routine practice conditions.

## Conclusions

Within the limitations of this *ex vivo* paired pre-post SEM study, repeated use of One RECI instruments increased the number of surface alterations compared with the factory-new baseline condition. Dull cutting edges and deformed flutes were the most frequent defects, whereas tip deformation was uncommon.

The ARI mode showed a tendency toward fewer surface defects than the manufacturer’s standard reciprocating motion. However, because the unadjusted ARI versus NOR comparison did not reach statistical significance and the intergroup sample size was limited, this finding should be interpreted cautiously. Further studies incorporating larger samples, dynamic torque monitoring, fatigue-to-failure testing, and blinded SEM assessment are needed to confirm whether adaptive reciprocation provides a clinically relevant protective effect on NiTi instrument surface integrity. 

## Supplementary Material

Supplementary 1

## Figures and Tables

**Table 1:** Table 1: Descriptive statistics of total surface defects by group.

**Group**	**Mean**	**Median**	**SD**	**Min**	**Max**
Factory (FAB)	0.15	0	0.48	0	3
Adaptive (ARI)	0.93	1	1.14	0	5
Normal (NOR)	1.63	2	1.47	0	5

SD: Standard deviation. Values represent total recorded defects after three uses (except FAB baseline condition).

**Table 2:** Table 2: Inferential statistical tests for group differences in surface defect counts.

**Test**	**Statistic**	*p value*
Shapiro-Wilk	W=0.669	*p*<.001
Kruskal-Wallis	χ²=38.22, df=2	*p*<.001
Wilcoxon (New vs Three Uses)	W=813.5	*p*<.001

p<.001 indicates statistically significant differences.

**Figure 1:**
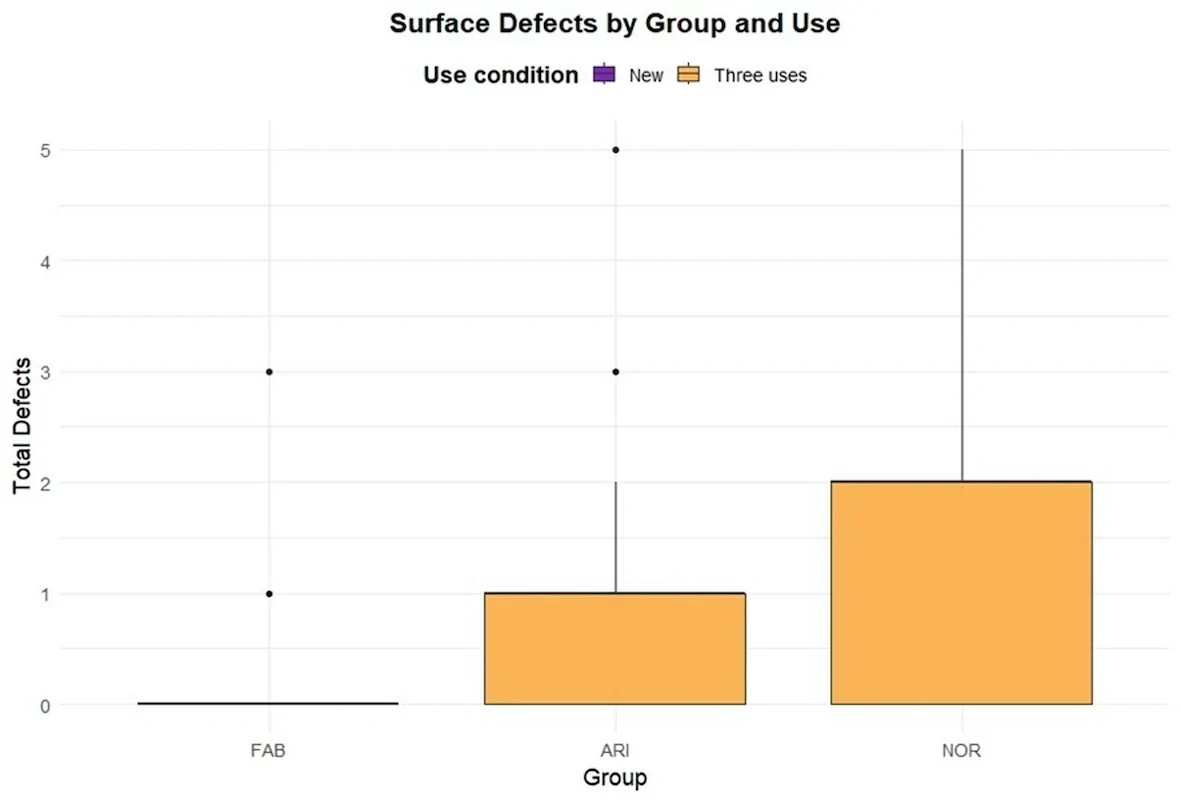
Fig. 1: Distribution of total surface defects per instrument according to experimental group and use condition. Instruments used three times exhibited markedly higher defect counts than new files, with the NOR group showing the greatest frequency of surface defects compared with the ARI and FAB groups.

**Figure 2:**
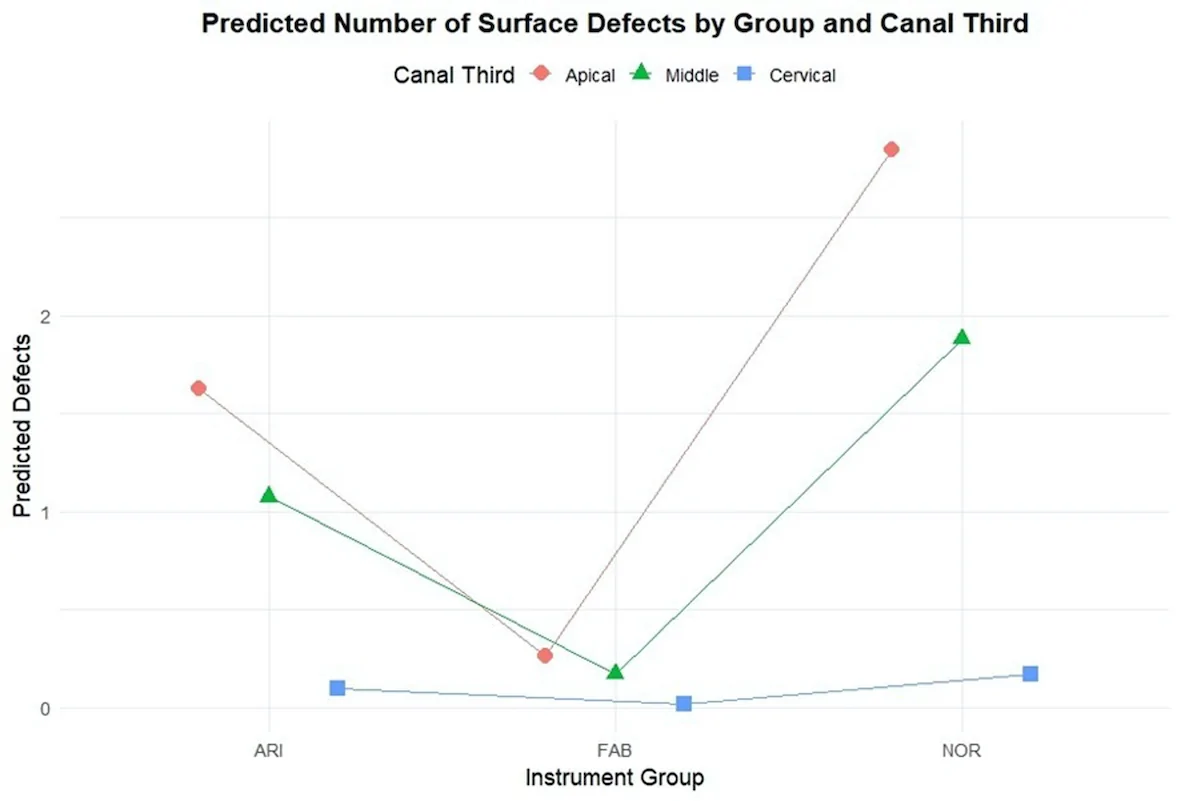
Fig. 2: Poisson regression-based predicted number of surface defects according to experimental group and canal third. The apical third showed the highest predicted defect counts, followed by the middle third, while the cervical third consistently exhibited fewer defects. Instruments in the NOR group demonstrated the highest expected number of surface defects.

**Figure 3:**
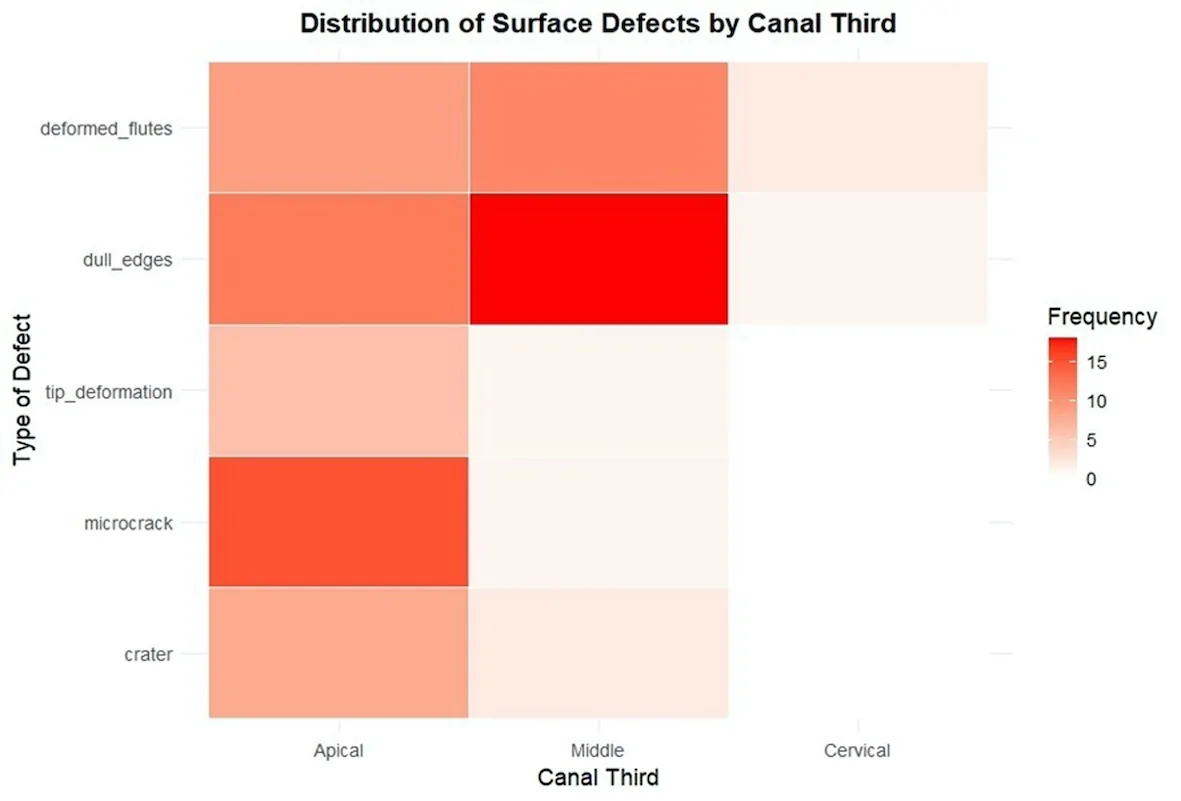
Fig. 3: Heatmap illustrating the distribution of surface defect types along the apical, middle, and cervical thirds of the instruments. Dull edges were most frequently observed in the middle third, whereas microcracks predominated in the apical third. The cervical third showed the lowest overall defect frequency.

**Figure 4:**
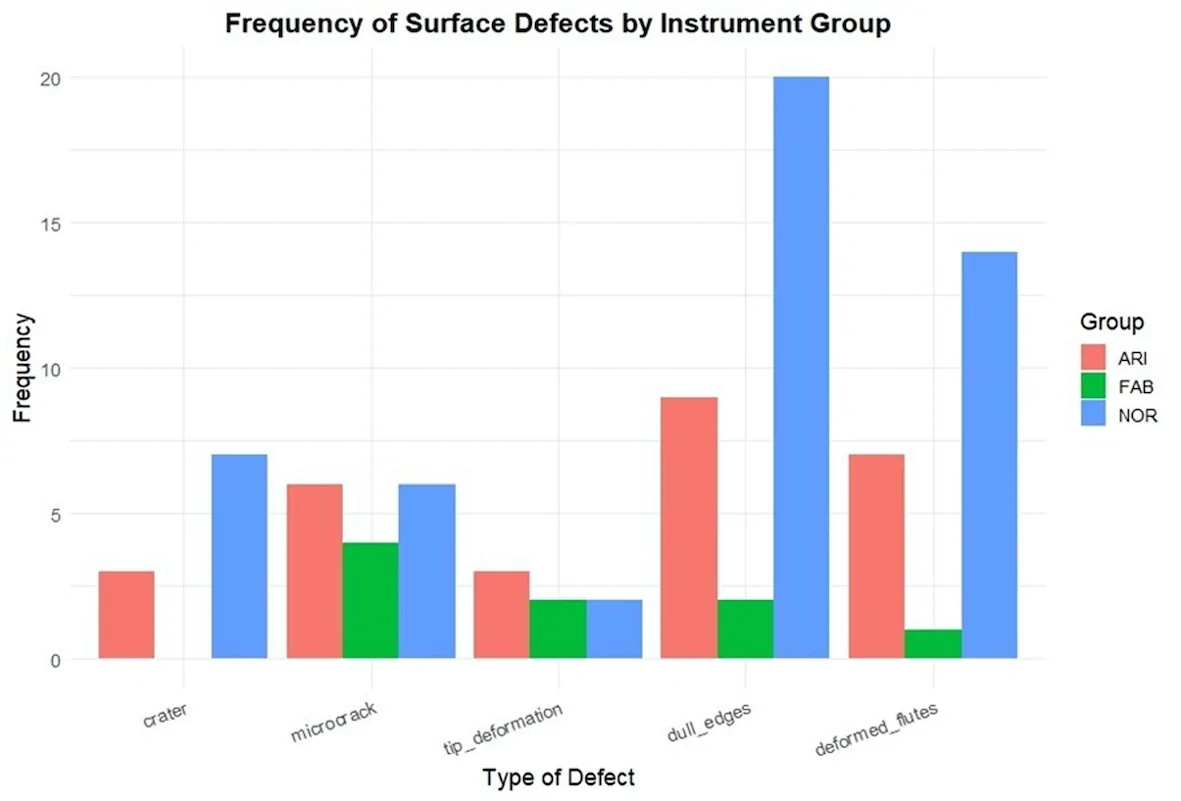
Fig. 4: Comparison of surface defect type frequency among experimental groups (FAB, ARI, and NOR). The NOR group exhibited the highest frequency of dull edges and deformed flutes, followed by the ARI group, whereas the FAB group showed minimal surface alterations.

## Data Availability

Declared none.
