# Supplementary material for: Surface alterations of the MicroMega One RECI reciprocating instrument under two kinematics: A scanning electron microscopy evaluation
Source: J Clin Exp Dent. 2026 Sep 15;18(10):e1281–9. doi: 10.4317/jced.63988 (PMC13739077; doi:10.4317/jced.63988)
Supplement: Supplementary file 1 [file jced-18-10-e1281-s001.pdf]

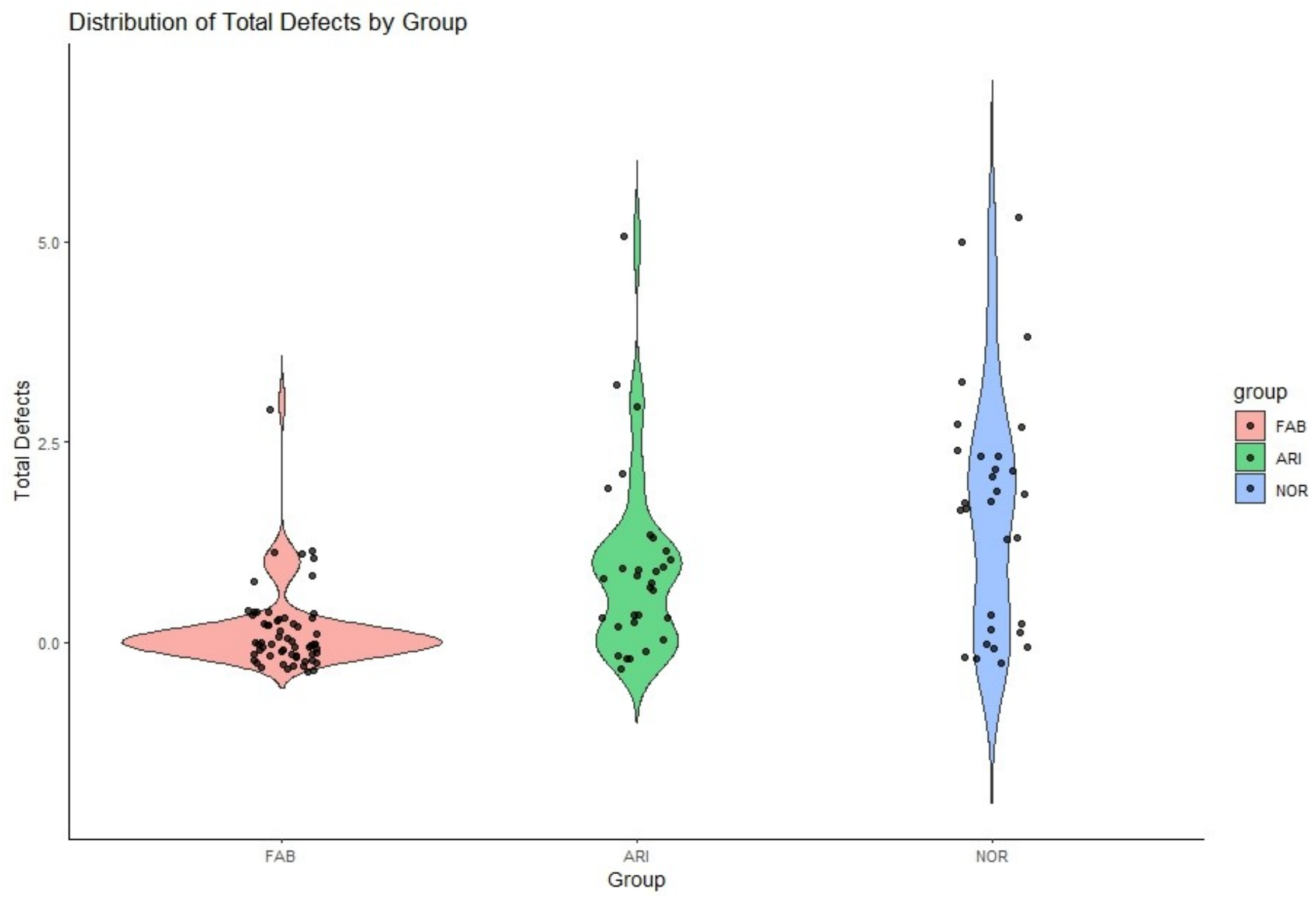

Figure 1.E.

Violin plot illustrating dispersion of total surface defect counts by experimental group. The FAB group presented minimal variability, while ARI and NOR demonstrated wider dispersion and higher concentration of instruments with multiple defects.

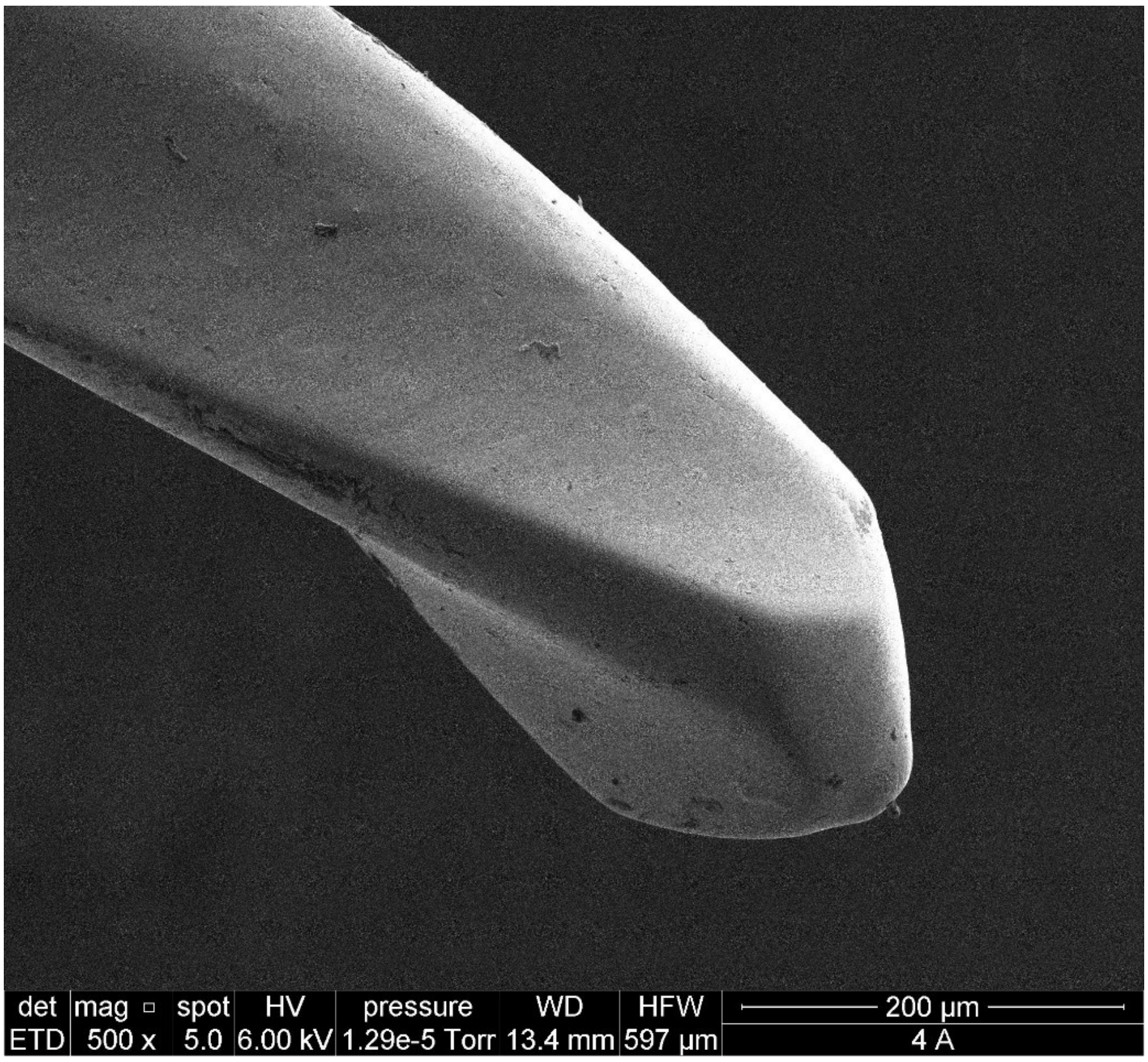

Figure 2.A. *Scanning electron microscopy image of new MicroMega One RECI (25.06) file at 500× magnification prior to use, showing a smooth surface without manufacturing defects.*

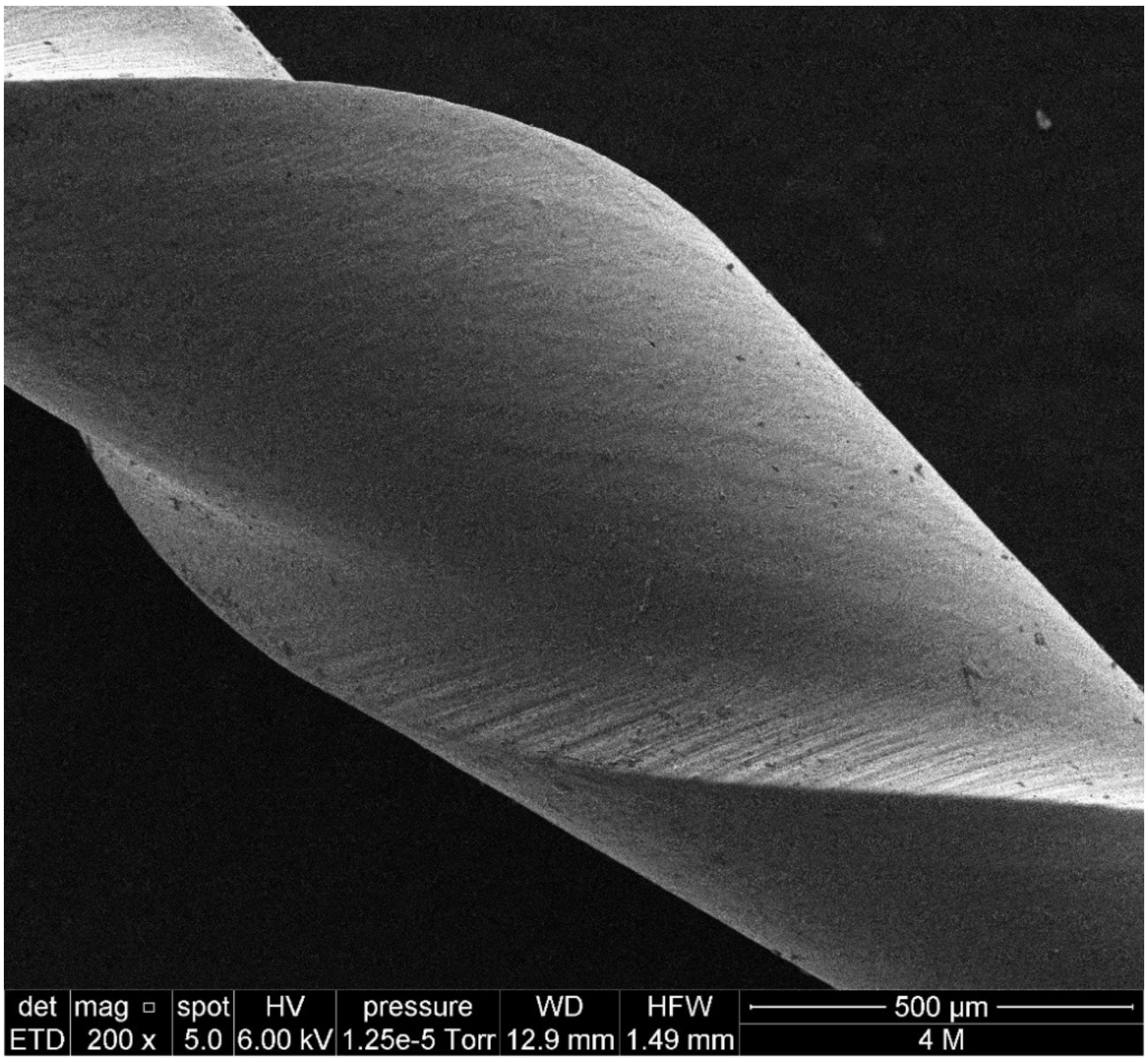

Figure 2.B. *Scanning electron image of new MicroMega One RECI file at 200× magnification before use, showing intact flutes and no structural factory defects.*

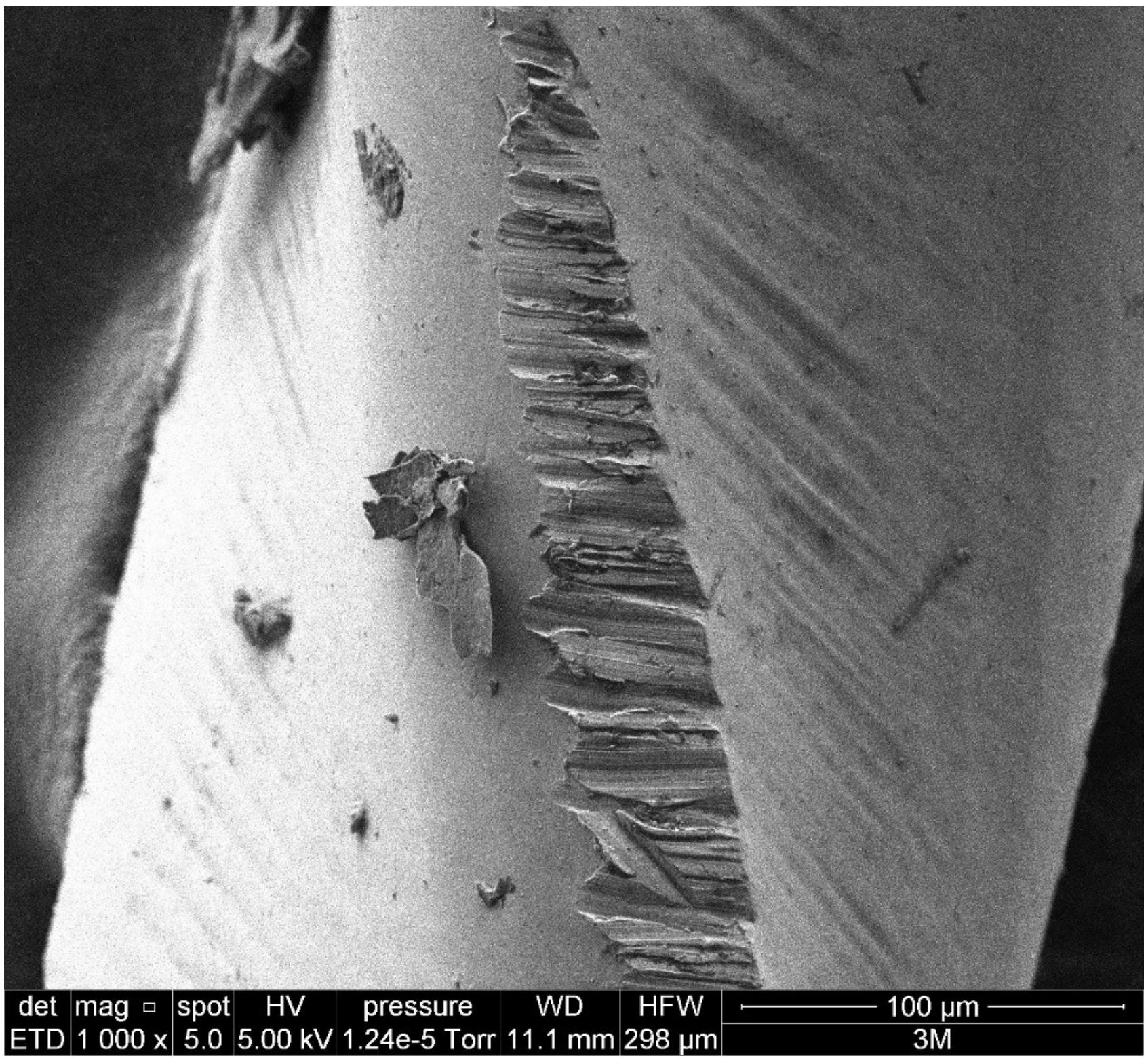

Figure 2.C. *Scanning Electron Image of One RECI file (NOR group) at 1000× magnification after three canal uses, showing surface defects such as craters, dull edges, and deformed flutes.*

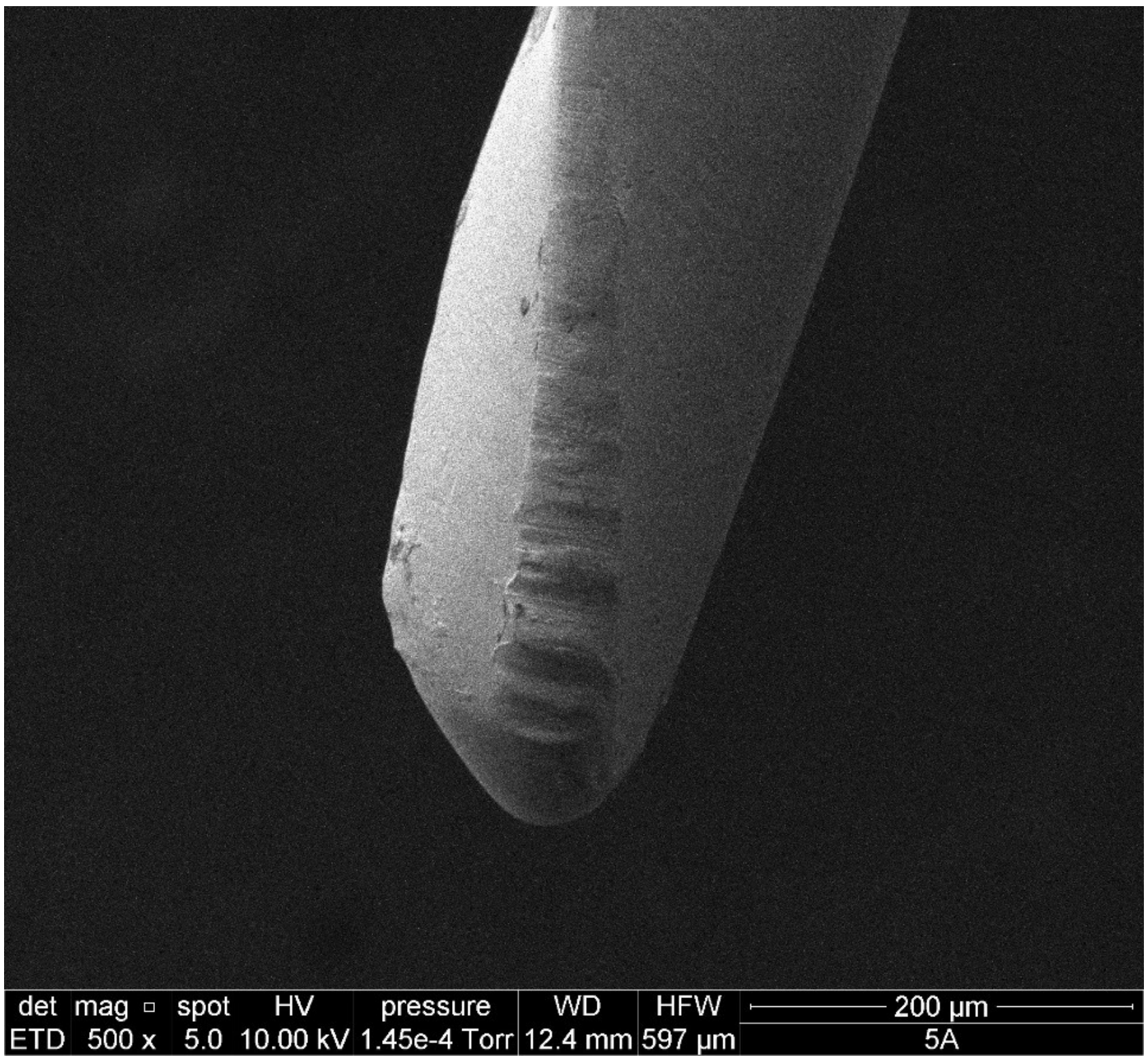

Figure 2.D. *Scanning Electron Image of One RECI file (NOR group) at 500× magnification after three canal uses, showing craters, tip deformation, dull edges, and deformed flutes.*

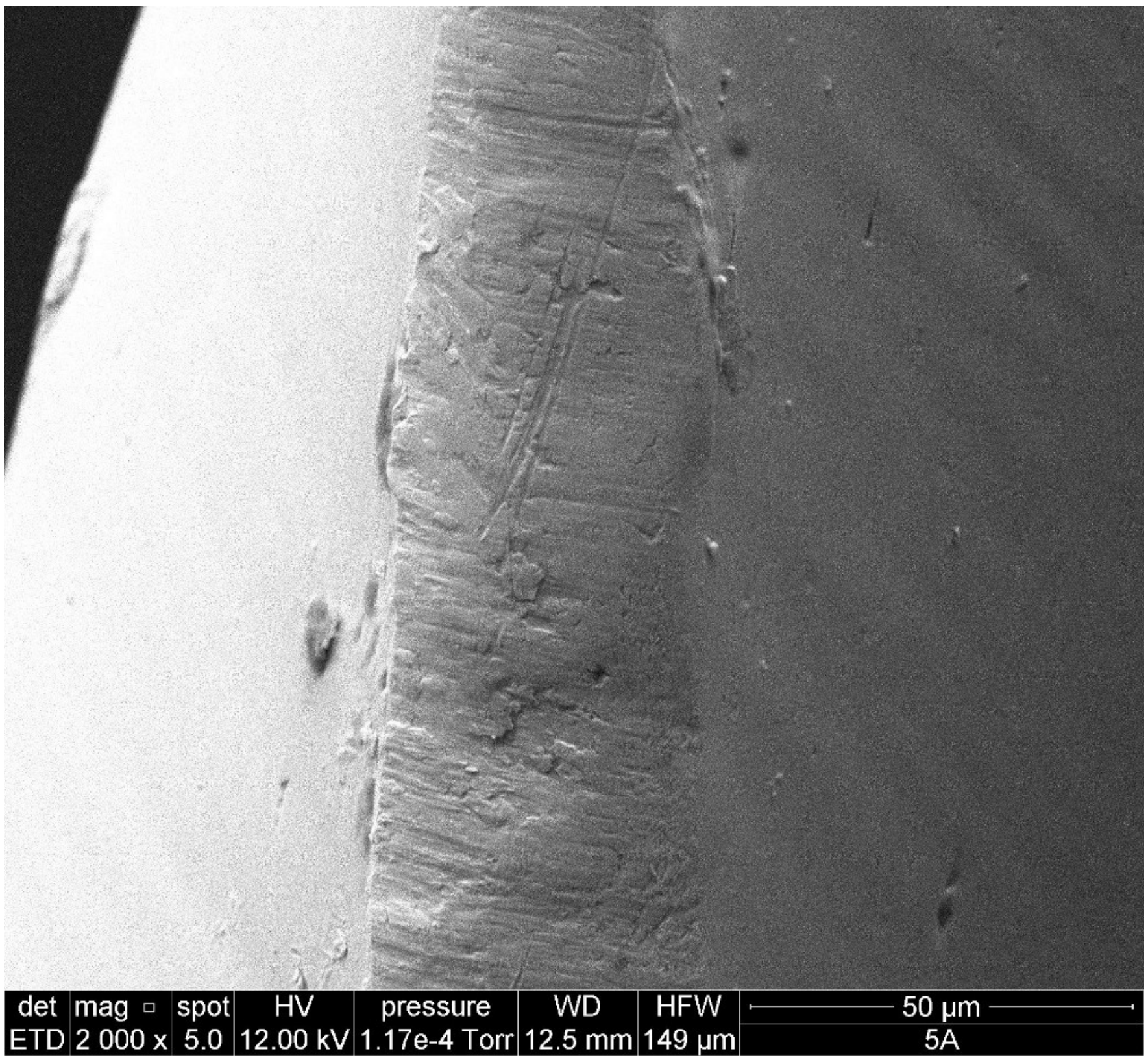

Figure 2.E. *Scanning Electron Image of One RECI file (NOR group) at 2000× magnification after three canal uses, showing craters, blunt tip, dull edges, and disrupted cutting edges.*

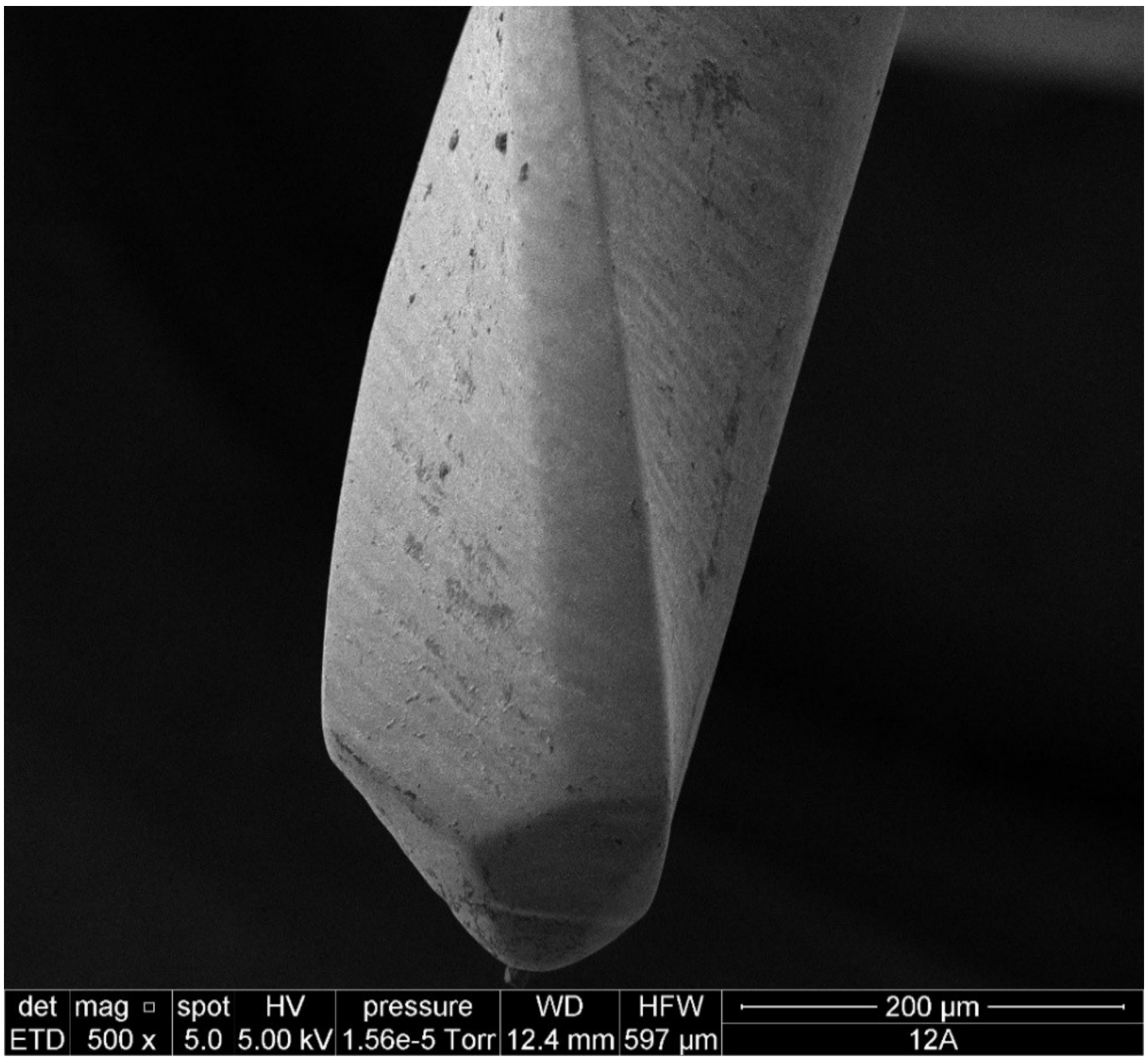

Figure 3.A. *Scanning Electron Image of One RECI file (ARI group) at 500× magnification after three canal uses, showing only a dull edge.*

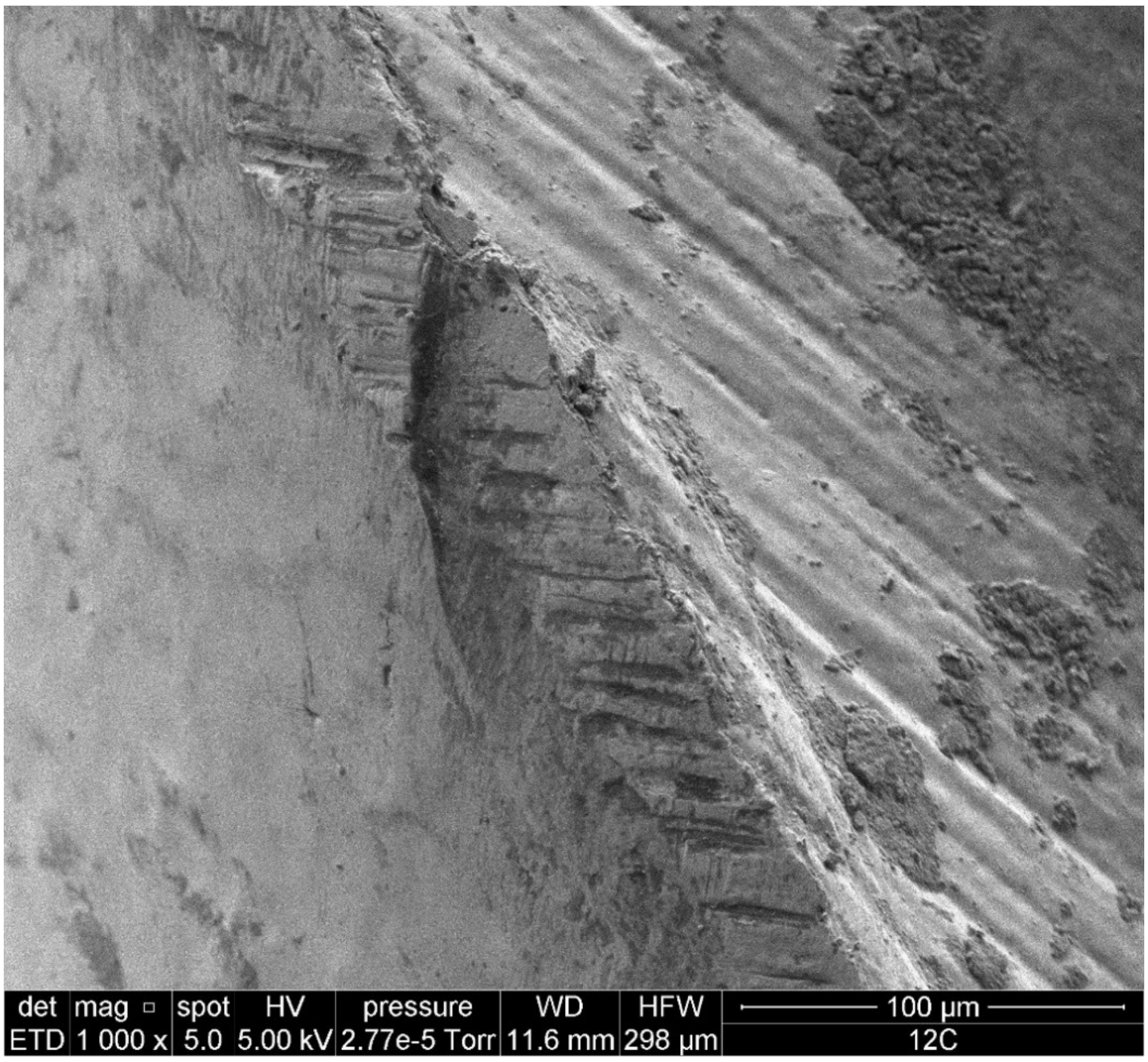

Figure 3.B. *Scanning Electron Image of One RECI file (ARI group) at 1000× magnification after three canal uses, showing only a disruption of the cutting edge.*

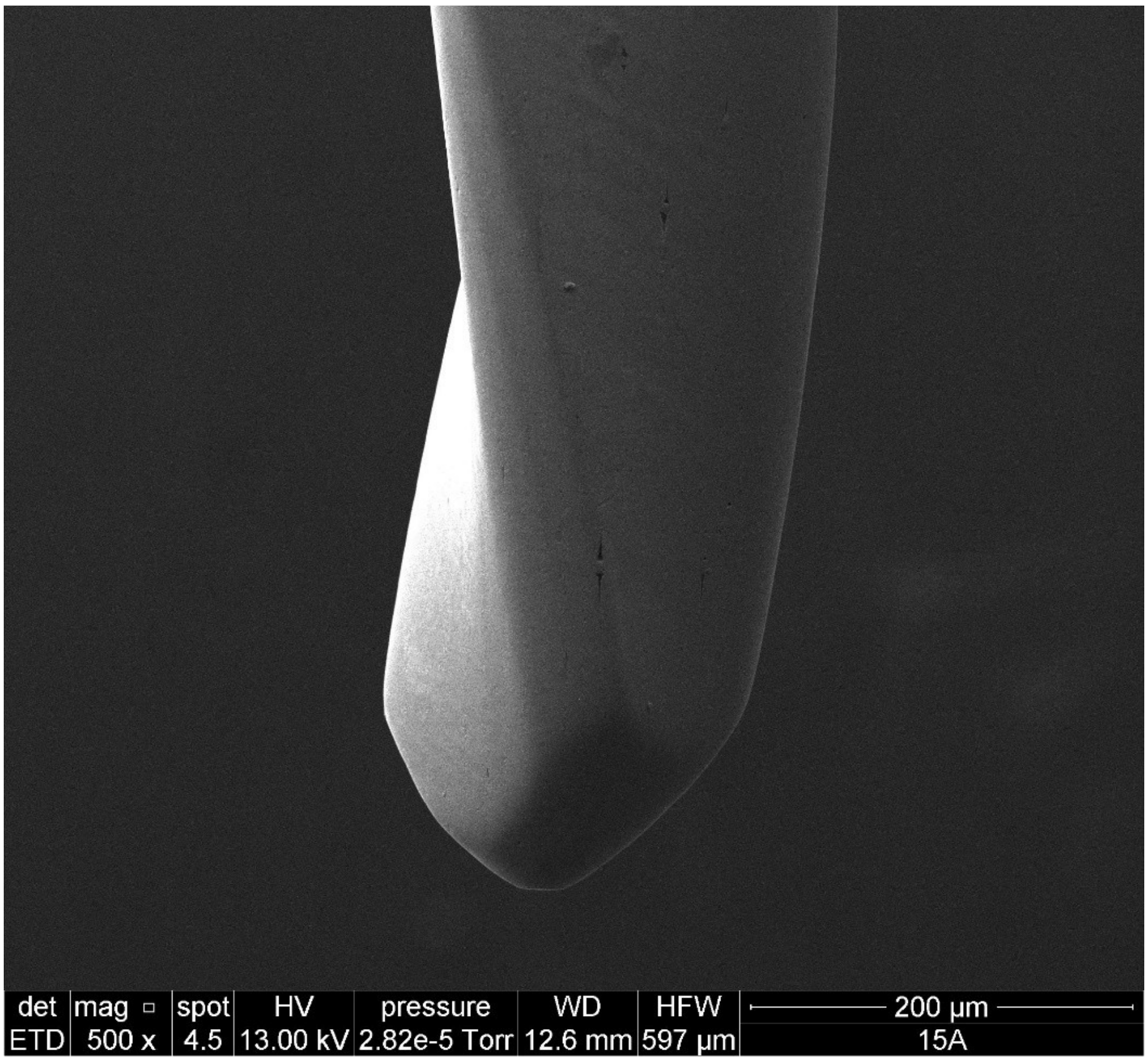

Figure 3.C. *Scanning Electron Image of One RECI file (ARI group) at 500× magnification after three canal uses, showing only a microcrack.*

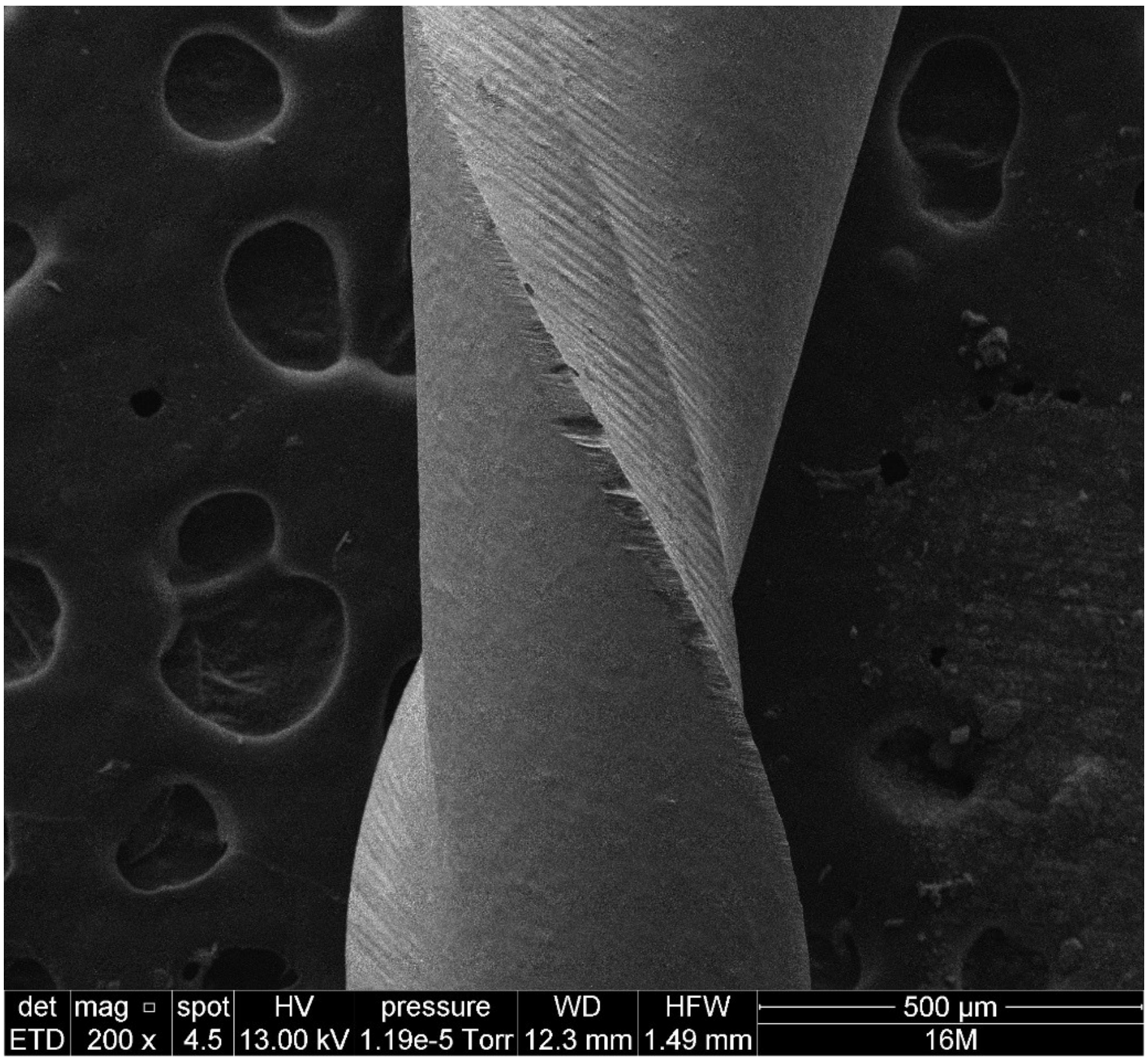

Figure 3.D. *Scanning Electron Image of One RECI file (ARI group) at 200× magnification after three canal uses, showing only disruption of cutting edges.*
